# Using nominal group technique to identify barriers and facilitators to preventing HIV using combination same-day pre-exposure prophylaxis and medications for opioid use disorder

**DOI:** 10.1186/s12954-022-00703-8

**Published:** 2022-10-28

**Authors:** William H. Eger, Frederick L. Altice, Jessica Lee, David Vlahov, Antoine Khati, Sydney Osborne, Jeffrey A. Wickersham, Terry Bohonnon, Lindsay Powell, Roman Shrestha

**Affiliations:** 1 Department of Internal Medicine, Section of Infectious Diseases, Yale School of Medicine, New Haven, CT USA; 2 Department of Allied Health Sciences, University of Connecticut, Storrs, CT USA; 3 Yale School of Nursing, New Haven, CT USA; 4 Institute for Collaboration on Health, Intervention, and Policy (InCHIP), University of Connecticut, 358 Mansfield Rd, Unit 1101, Storrs, CT 06269 USA

**Keywords:** Nominal group technique, People who inject drugs (PWID), Pre-exposure prophylaxis (PrEP), Opioid agonist treatments, Implementation science

## Abstract

**Background:**

Preventing HIV transmission among people who inject drugs (PWID) is a key element of the US Ending the HIV Epidemic strategy and includes both pre-exposure prophylaxis (PrEP) and medications for opioid use disorder (MOUD). While both lead to decreases in HIV transmission, MOUD has other social and health benefits; meanwhile, PrEP has additional HIV prevention advantages from sexual risk and the injection of stimulants. However, these medications are often prescribed in different settings and require multiple visits before initiation. Strategies to integrate these services (i.e., co-prescription) and offer same-day prescriptions may reduce demands on patients who could benefit from them.

**Methods:**

Nominal group technique, a consensus method that rapidly generates and ranks responses, was used to ascertain barriers and solutions for same-day delivery of PrEP and MOUD as an integrated approach among PWID (*n* = 14) and clinical (*n* = 9) stakeholders. The qualitative portion of the discussion generated themes for analysis, and the ranks of the proposed barriers and solutions to the program are presented.

**Results:**

The top three barriers among PWID to getting a same-day prescription for both PrEP and MOUD were (1) instability of insurance (e.g., insurance lapses); (2) access to a local prescriber; and (3) client-level implementation factors, such as lack of personal motivation. Among clinical stakeholders, the three greatest challenges were (1) time constraints on providers; (2) logistics (e.g., coordination between providers and labs); and (3) availability of providers who can prescribe both medications. Potential solutions identified by both stakeholders included pharmacy delivery of the medications, coordinated care between providers and health care systems (e.g., case management), and efficiencies in clinical care (e.g., clinical checklists), among others.

**Conclusions:**

Implementing and sustaining a combined PrEP and MOUD strategy will require co-training providers on both medications while creating efficiencies in systems of care and innovations that encourage and retain PWID in care. Pilot testing the co-prescribing of PrEP and MOUD with quality performance improvement is a step toward new practice models.

**Supplementary Information:**

The online version contains supplementary material available at 10.1186/s12954-022-00703-8.

## Background

People who inject drugs (PWID) are a key population that is prioritized by the US strategy to End the HIV Epidemic (EHE), which aims to utilize a toolkit of evidence-based interventions to prevent new infections [1]. While HIV incidence has dropped among PWID over the past few decades, there has been a surge of new cases due to outbreaks in counties with suboptimal prevention endeavors [2].

Because PWID have both sexual- and injection-related risk behaviors for HIV, a single prevention strategy alone may not be sufficient [3, 4]. The HIV prevention toolkit for PWID includes four evidence-based practices: pre-exposure prophylaxis (PrEP), medication for opioid use disorder (MOUD) to treat opioid use disorder, syringe services programs (SSPs), and HIV treatment as prevention (TasP) [2]. While each component of the toolkit has the potential to complement the other, they are often delivered in siloed systems that limit their impact. For example, SSPs provide PWID with access to harm reduction services but are limited geographically and often lack the resources to deliver MOUD. Compared to MOUD, SSPs and PrEP have the benefit of preventing HIV among people who inject stimulants. At the same time, MOUD alone works to reduce social (e.g., employment, incarceration, etc.) and medical (e.g., overdose, depressive symptoms, engagement in care, etc.) harms from opioid injection and addiction [5].

Scale-up of PrEP and MOUD among PWID has been limited and is burdened by requiring a prescription often delivered in different settings by clinicians with varying areas of expertise [6]. When treatment is not integrated, it creates excess demands on patients to navigate different systems and clinicians, which can have challenges for PWID, whose lives are often unstable and face stigma that interferes with their engagement in care [7].

The systems of care for prescribing PrEP and MOUD are seemingly more complicated than they need to be. With PrEP, for example, most providers may require multiple visits prior to prescription, creating substantial demands on patients, clinicians, and the health care system [8]. The distilled elements minimally include a risk assessment for eligibility, laboratory testing, and prescription. Though the US Centers for Disease Control (CDC) now recommends same-day access to PrEP for individuals at high risk for contracting HIV, hesitation toward this strategy has been observed for populations like PWID, who are viewed as “unstable” [9]. Similarly, the prescription of MOUD has its own set of requirements [10], some of which are regulated by the Drug Enforcement Agency (DEA). Not to mention that requirements differ for methadone and buprenorphine (with fewer restrictions for buprenorphine), which can further complicate patient care [11]. During the onset of the COVID-19 pandemic, however, restrictions for MOUD were substantially decreased, with no in-person visit requirements for buprenorphine. The same is now true for PrEP aside from laboratory testing.

The “Test and Treat” (TnT) strategy provided evidence for safe and effective same-day antiretroviral therapy (ART) prescription and management. This means that patients receive a prescription for medication on their first doctor visit without waiting for a follow-up visit (usually to review laboratory test results before prescription) [12–14]. PrEP and MOUD (specifically buprenorphine), like contemporary ART, have emerged as safe and effective treatments requiring minimal monitoring [15, 16]. While effectiveness studies of TnT for HIV in PWID and same-day prevention by co-prescribing PrEP and MOUD remain unreported to date, increasing studies have demonstrated the efficacy of an integrated model of health care survey delivery. For example, a recent study using bundled screen, evaluate and treat (SET) procedures to minimize the demands of HCV treatment among SSP clients and providers reported that 94.5% of actively injecting PWID treated achieved cure. Among this group, the majority were co-prescribed MOUD, which likely benefited HCV outcomes, had further benefits of stabilizing patients, and simultaneously improved viral suppression levels for people with HIV [17]. To prepare for an integrated approach to provide same-day PrEP and MOUD prescribing strategy collocated within an SSP, we inquired about barriers and facilitators to this strategy among PWID and relevant clinical stakeholders, including clinicians.

## Methods

### Participants and settings

Between February and August 2021, we recruited 23 respondents (14 PWID and 9 clinical stakeholders). PWID were recruited through posted advertisements and word-of-mouth at a syringe services program (SSP) in New Haven, Connecticut. Inclusion criteria for PWID included being 18 years or older, self-reported HIV-negative, having injected drugs within the past month, and having a substantial ongoing risk of HIV infection (e.g., shared needles or condomless sex in the last month). Clinical stakeholders included Advanced Practice Clinicians (APCs) with “X” waivers to prescribe buprenorphine, patient navigators, and addiction treatment counselors who worked at federally qualified health centers (FQHCs) and were involved in providing HIV-related and/or addiction treatment services to the target population. FQHCs included addiction treatment centers (ATCs) or SSPs. Clinical stakeholders were recruited through email to relevant organizations throughout New Haven.

### Procedures

We utilized the nominal group technique (NGT), a mixed-methods focus group strategy, to understand the barriers and facilitators to same-day access to PrEP and MOUD. NGT is a structured mixed-methods strategy that rapidly generates and prioritizes solutions [18]. NGT was selected because it creates quantitative estimates (rank-ordering) combined with in-depth qualitative information that can be implemented relatively quickly. The advantage of NGT compared to traditional focus groups is inclusivity by facilitating equal participation by all group members despite power imbalances. A silent generation of responses, followed by a round-robin listing of responses, is followed by a discussion of the responses with independent voting by each member to ensure each individual’s input. Voting and discussion allow the aggregation of individual judgments into group conclusions.

Five NGT sessions (two among PWID and three among clinical stakeholders) were conducted online using a video conferencing platform (WebEx), and each session lasted about 60 min. An average of four participants, ranging from three to nine per session, were included in each NGT session. A trained facilitator led the NGT session, while a co-facilitator took notes, recorded non-verbal cues, and wrote/typed responses on a ‘whiteboard.’ Each session was audio-recorded with the participants’ permission and was transcribed. Each participant provided verbal consent before starting the session and was compensated with a $25 gift card for their participation. The study protocol was approved by the Institutional Review Boards at Yale University and the University of Connecticut.

Questions for the NGT sessions were developed after a literature review and consultation with experts in HIV prevention, addiction treatment, and NGT methodology. To contextualize the questions and the intended intervention for our participants, we started each session with a brief overview of PrEP and MOUD, procedures for initiating PrEP and MOUD, and the session's goal. An example of our interview guide used for the PWID sessions is outlined in the Additional file 1: Appendix. Following our introduction, the following questions were addressed in each session:

- If a person wanted to start on PrEP and MOUD (like methadone or buprenorphine), what things might get in the way of getting it prescribed to them on the same day?
- Based on the top three priorities identified in the last question [these were listed for review], what types of resources or support do you think are required to develop a program that provides both PrEP and MOUD on the same day?

After each question, participants silently generated unique ideas for three minutes, either in writing or quietly to themselves. Then, using the ‘round-robin’ elicitation process, each person contributed a single idea recorded visually on a whiteboard. Additional rounds were completed until responses were saturated. Participants then engaged in group discussions to clarify and evaluate ideas, and items were grouped by consensus, with duplicate items removed. Next, individuals voted to prioritize items; each participant had three votes to cast in any combination of the listed item(s) they deemed most important (e.g., three votes on one item, one vote per each of the three items, etc.). Votes were immediately tallied and ranked (based on the total number of votes). The facilitator led a final discussion to review the participants' results and see if they had face validity with the group. Additionally, each participant provided demographic information via a brief Qualtrics survey.

### Analytical plan

All generated responses to questions were recorded, and votes were tabulated per item per group (i.e., PWID and clinical stakeholders). We identified the highest-ranking responses per question, pooled them across groups, and organized them by question. Post hoc analysis of the audio recordings and detailed notes collected during the NGT sessions were used to establish the major themes presented. Transcripts from the group sessions were reviewed and coded by W.H.E. and J.L. and were analyzed using thematic analysis. If coding was discordant, a conflict resolution of the coding ensued, and where consensus was not achieved, a third person broke the tie (a rare event). Responses from each session and qualitative data gathered during the group discussions were then analyzed, and the results for the identified items were recorded to arrive at a final score for the prioritization process. The thematic framework categorized the highest-ranking responses, and the transcripts contextualized these priorities [19].

Following recommendations for analyzing NGT session data across multiple groups, we consolidated raw ranking data, then conducted iterative rounds of thematic coding of both responses and transcripts [20]. A combination of the raw ranking data and the thematic coding was used to create the major themes for our results, which included specific barriers or solutions posed by our participants. Using the top-ranked items from each session, two authors, W.H.E. and A.K., synthesized the results across sessions and tallied votes accordingly. Since each member received an equal number of votes, and all votes were consolidated at the end of each session and across sessions, the overall themes presented represent the raw and balanced votes achieved for that item across all sessions with those stakeholder members.

## Results

### Participant characteristics

Table 1 shows the sociodemographic and treatment-related characteristics of the sample. Among PWID, the majority of participants were White, male, had a high school education or less, and earned less than $9,999 annually (from both legal and illegal sources). Among PWID, 14.3% were prescribed PrEP, and 57.1% were prescribed MOUD. Most clinical stakeholders were White and included four APCs, three physicians, one patient care associate, and one addiction treatment counselor. The majority of clinical stakeholders were involved in providing traditional PrEP- (88.9%) and MOUD-related (77.8%) services.

**Table 1:** Characteristics of participants (N = 23) †

	**PWID**		**Clinical Stakeholders**	
	N	%^‡^	N	%
**Total**	14	100	9	100
Median age, years	43	–	40	–
Gender				
Male	8	57.1	2	22.2
Female	6	42.9	7	77.8
Race and ethnicity				
White	8	57.1	5	55.6
Hispanic/Latinx	4	28.6	3	33.3
Black/African American	2	14.3	1	11.1
Education				
High school or lower	11	78.6	0	00.0
Some college or higher	3	21.4	9	100.0
Income				
Less than $9,999	11	78.6	–	–
Greater than $10,000	1	7.1	–	–
Employment status				
Employed for wages	4	28.6	–	–
Unemployed or disabled	10	71.4	–	–
Currently prescribed PrEP				
No	12	85.7	–	–
Yes	2	14.3	–	–
Currently prescribed MOUD				
No	6	42.9	–	–
Yes	8	57.1	–	–
Directly involved with PrEP services				
No	–	–	1	11.1
Yes	–	–	8	88.9
Directly involved with MOUD services				
No	–	–	2	22.2
Yes	–	–	7	77.8
Profession				
Advanced Practice Registered Nurse	–	–	4	44.4
Physician	–	–	3	33.3
Patient Care Associate	–	–	1	11.1
Addiction Treatment Counselor	–	–	1	11.1

*PWID* people who inject drugs; *PrEP* pre-exposure prophylaxis for HIV; *MOUD* medication for opioid use disorder

^†^Table values are n and (%) for categorical variables and median for continuous variables

^‡^Numbers may not sum to total due to missing data, and percentages may not sum to 100% due to rounding

### NGT sessions with PWID

#### Barriers to implementing same-day prescription of PrEP and MOUD

Potential barriers to prescribing same-day access to PrEP and MOUD are categorized into four themes (Table 2): (1) social determinants of health barriers; (2) structural-level implementation factors; (3) client-level implementation factors; and (4) knowledge barriers.

**Table 2:** Potential barriers for implementing same-day prescription of PrEP and MOUD among PWID and Clinical Stakeholders (N = 23)

“If a person wanted to start on PrEP and MOUD (like methadone or buprenorphine), what kinds of things might get in the way of getting it prescribed to them on the same day?”	Voting results
Persons who inject drugs (n = 14)	42
Social determinants of health barriers	22
Lack of, or unstable insurance	11
Lack of transportation to appointments and pharmacy	6
Family support (e.g., access to childcare)	2
Lack of communication tools (i.e., phone or Internet)	2
Too costly/financially unstable	1
Structural-level implementation factors	8
Access to a provider	4
Stigma of ‘drug use’	2
Mistrust in clinicians	2
Client-level implementation factors	8
The process is too time-consuming	2
Need to attend different clinics	2
Lack of interest/motivation to take the medications	2
Cultural beliefs or religious obligations	1
Actively using drugs or feeling ‘dope sick’	1
Knowledge barriers	4
Fear of side effects and mixing of medications	3
Lack of information about the two medications	1
Clinical Stakeholders (n = 9)	27
Time constraints on providers for clinical assessments	7
Logistics (e.g., coordination between providers and labs)	6
Availability of providers that can prescribe both medications	5
Unreliable population (e.g., complications after getting prescription)	4
Hesitancy of providers (e.g., due to getting lab results after the prescription process)	3
Transportation challenges of the participants	1
COVID-19 (e.g., following guidelines; future procedures; screening protocols)	1

*PrEP* pre-exposure prophylaxis for HIV; *MOUD* medication for opioid use disorder

More than half of the total votes were cast toward barriers related to the social determinants of health, with most participants pointing toward a lack of or instability of their insurance. Lack of transportation to appointments and the pharmacy, barriers related to family support (e.g., access to childcare), lack of communication tools (i.e., phone or Internet), and financial barriers were also highlighted.

Structural factors, which included access to a local PrEP or MOUD provider, stigma related to ‘drug use,’ and mistrust in physicians, also posed hindrances to receiving same-day access to PrEP and MOUD:

“We need more doctors to see each patient on a daily basis. You’re saying ‘walk out the door right now’ but I can’t walk out the door and just call a private number to a doctor and say ‘look, I wanna see you right now.’”

– 42-year-old (yo) female on MOUD

Despite being a challenge among a majority of PWID, one of the participants still noted their surprise when ‘access to doctors’ did not receive a larger number of votes in their NGT session:

“I mean, okay, you got nine people right here. If we all wanted both pills at one time, the doctor can’t see all of us today.”

– 42 yo female on MOUD

This response prompted others to join in on the conversation, with five participants in the session stating that they would likely have changed their vote upon further consideration. Further, some participants perceived that they would be discriminated against due to their drug use, reflected by one participant stating:

“Stigma of being a drug user… you don’t really put that out in public.”

– 43 yo male not on PrEP or MOUD

“I remember trying to sign up for Suboxone and [the doctor] actually told me that I am not a good candidate for [it] and that I should actually get on Methadone because I had too high a chance of relapsing… Who the fuck are you to tell me that I have too high a chance of relapsing?”

– 31 yo female not on PrEP or MOUD

Other commonly reported challenges were client-level implementation factors, evenly split across several items. PWID expressed that the process of same-day access to PrEP and MOUD might be too time-consuming for them, especially if they needed to attend different clinics for PrEP and MOUD. Some participants specifically mentioned that this might undermine their ability to access same-day services for both medications and reduce their interest or motivation to engage fully. One participant noted that cultural beliefs, like the perception that medications should not be used for treatment, could hinder same-day access to PrEP and MOUD, but this was not a widely held position. One community member stated that actively using drugs or experiencing symptoms of withdrawal (‘dope sick’) from opioids while preparing to be inducted onto buprenorphine posed an obstacle toward same-day access to PrEP and MOUD for them too:

“I feel like if you’re dope sick, nobody’s doing anything.”

– 31 yo female not on PrEP or MOUD

Last, inadequate knowledge was stated as a key barrier to same-day treatment, with concerns about potential additive side effects from two different medications or concerns about the “mixing” of medications and subsequent potential harm. One participant stated:

“I’m more so thinking about what it’ll do to [me], the side effects. I never took PrEP, so what will it do to me? How would it affect me? I really would need to know more about the drug.” – 50 yo female not on PrEP or MOUD

#### Strategies to support the implementation of same-day prescription of PrEP and MOUD

After repeating the top three barriers, participants discussed the potential solutions for receiving same-day access to PrEP and MOUD, which are listed in Table 3. The two most commonly stated facilitators were home delivery and access to more clinicians or locations that could prescribe both medications simultaneously. Most PWID said that home delivery of medications would be essential to implementing this program, given their concerns about transportation in general, including to the pharmacy. Multiple participants exclaimed their desire for at-home delivery:

“Home delivery would be the bomb!”

– 51 yo female on MOUD

“That’s the best thing possible.”

– 43 yo female on MOUD

**Table 3:** Potential solutions for implementing same-day access to PrEP and MOUD among PWID and Clinical Stakeholders (N = 23)

“Based on the top three priorities identified in the last question, what types of resources or support do you think are required for developing a program that provides both PrEP and MOUD on the same day?”	Voting results
Persons who inject drugs (n = 14)	42
Home delivery by pharmacy	16
Access to more clinicians (and locations) that can prescribe both medications	10
Easy access to information (e.g., social media; WhatsApp)	6
Access to transportation (e.g., bus passes; pre-paid taxis)	3
Concrete appointment times	3
Access to telehealth	2
Health insurance assistance	2
Clinical Stakeholders (n = 9)	27
Coordinated care between providers and systems (e.g., case management)	7
Expedited system processing and coordination (e.g., clinical templates, checklists, smart phrases in EMR system; rapid testing for HIV)	6
Designated appointment slots and time allocated for these types of visits	5
Access to transportation for participants (e.g., bus passes; pre-paid taxis)	4
Standardization of treatment processes across systems	3
Provider education (e.g., mentorship and case reviews)	2

*PrEP* pre-exposure prophylaxis for HIV; *MOUD* medication for opioid use disorder; *EMR* electronic medical record

Access to more providers and locations that can prescribe both medications at the same time was the second most common facilitator stated for successful program implementation. For one client who was currently prescribed MOUD, they stated that getting both medications as ‘a one-stop shop’ would be an important solution:

“Ya know, killing two birds, one stone. Getting them in the same spot…”

– 46 yo male on MOUD

Other important but less commonly cited facilitators for same-day PrEP/MOUD included improved access to information through social media or other online platforms (e.g., apps) to identify settings to get both information and services. Also, increasing access to transportation by providing free transportation through bus passes or prepaid taxi services and access to telehealth were specifically mentioned as solutions to overcome transportation challenges. Ensuring concrete appointment times and assistance with gaining health insurance was also important facilitators for this program. In the case of health insurance assistance, it was explicitly recommended that case managers would be ideal:

“When you go to sign up… they offer all the things… they offer PrEP… and if you don’t have health insurance, they walk you through the paperwork… hav[ing] counselors that show you how to set it up and walk through [the process].”

– 31 yo female not on PrEP or MOUD

### NGT sessions with clinical stakeholders

#### Barriers to implementing same-day prescription of PrEP and MOUD

The most commonly cited barrier to same-day PrEP/MOUD among clinical stakeholders was time constraints on providers for clinical assessments (Table 2), followed by logistics to efficiently complete their work and a sufficient number of providers who can prescribe the medications. For the barrier of time constraints, one APC stated:

“We’re not just doing PrEP and vaccinating, we’re doing everything else, and a lot of providers are kind of full to capacity. Even finding an open slot to do an appointment like this can be difficult on a dime, especially an appointment like this for like intake for Suboxone alone. It’s an engaging kind of visit, but for me, it’s just kind of finding a slot in my schedule.”

Other clinical stakeholders were concerned about logistical barriers that make their job too complex, like coordination of clinical evaluations that include history, examinations, and review of laboratory results. Next, our clinical stakeholders perceived that there were too few providers that could provide both medications, in part related to the treatment of two diverse conditions without adequate training:

“These are complex patients, and… if you're working with a whole lot of providers and only one or two of you are doing this kind of thing, then you're setting yourself up to have a much more complex panel because each one of these patients is going to wind up being a load of extra work for you and your nurse and your medical assistant. And you know, you are self-selecting for hardship, right?”

Clinicians expressed that the unreliability of the target population (i.e., PWID) in their ability to be contacted might prevent them from feeling confident in providing same-day PrEP and MOUD, as complications could arise after the patient receives the medications. Some clinical providers expressed hesitancy to prescribe PrEP and MOUD on the same day, as the immediate prescription process could require prescribing before getting the official results of laboratory tests. These two barriers strike a staggering synergy when explained by one of the APCs:

“I wouldn’t do that on an initial patient because you may never see them again, and God forbid you have something abnormal and you can't find them.”

Transportation challenges of PWID, as well as challenges posed by COVID-19, such as screening protocols and other safety precautions, were also emergent themes.

#### Strategies to support the implementation of same-day prescription of PrEP and MOUD

Among clinical stakeholders, coordinated care between providers within a health care system, such as having access to an assistant to review laboratories or provide case management, was the most common solution. Other commonly reported solutions were related to strategies that could help expedite the processing and coordination of care within a health care system, like integrated patient templates and flow sheets, the use of smart phrases in an Electronic Medical Record (EMR), or rapid testing for HIV. Designated appointment slots and time allocated for the same-day visits were also posed as a top solution to overcoming barriers related to the same-day prescription of PrEP and MOUD. Increasing patient access to transportation (e.g., bus passes; prepaid taxis) received four votes among clinical stakeholders. Standardizing treatment processes across systems and provider education through guided mentorship and case reviews received three and two votes, respectively (Table 3).

## Discussion

This study illustrates critical barriers and potential facilitators to inform the development of an integrated HIV prevention program, which incorporates same-day access to PrEP and MOUD. Our findings show that the future implementation of such an integrated HIV approach among PWID will likely be influenced by several factors, including the accessibility of the program, lack of providers to prescribe the needed medications, difficulties integrating services, and client-level implementation factors, among others. The findings support the need for a differentiated model of HIV service delivery to address specific challenges experienced by this vulnerable population, which might include a prescription for PrEP and/or MOUD, integrated patient templates, and a diverse clinical team to assist with care coordination. The use of a differentiated service delivery model focuses on patient-centered care, decreasing burdens for clinic visits and treatment, and reducing overall costs on the health care system, which may also involve collocating services [21].

Consistent with prior findings [22, 23], the social determinants of health (SDOH), such as transportation challenges, lack of or under-insurance, and access to providers, were the most salient perceived barriers to receiving same-day PrEP and MOUD among PWID. Logistical challenges, like access to transportation, among PWID are well documented [24]. The integration of PrEP and MOUD helps address some of these logistical barriers through the co-location of care; however, the demand for patients to attend appointments and receive their prescriptions remains unresolved in this model. As identified by our stakeholders, other solutions to this issue could include the delivery of medications by a pharmacy and providing transportation vouchers (i.e., bus passes or pre-paid taxis) to patients.

As shown through our discussions, telehealth could also limit the requirement for patients to attend in-person appointments. As was observed during the COVID-19 pandemic, telehealth is largely feasible for PrEP and MOUD delivery (especially buprenorphine) among PWID [25, 26]. Additionally, where possible, having this type of program in an ATC or SSP could prove beneficial in addressing specific concerns over access and patient demands [23, 27, 28]. The availability of low-threshold options for access to care (i.e., combined MOUD and PrEP) will help address some of the specific challenges experienced by PWID with opioid use disorder (OUD).

Another consideration to address integrated service delivery was demonstrated during the COVID-19 pandemic for clients at SSP sites where there was co-screening for HCV, HIV and OUD. Within that integrated service delivery model, laboratory screening was reflexed using an algorithm to evaluate each condition and determine eligibility for treatment. Such simplification of screening and evaluation could markedly reduce demands on clinicians and patients with busy schedules while maintaining equal or better treatment effects [17]. Differentiated models of care such as this might be modified and prove beneficial for combined PrEP and MOUD care delivery.

Innovations in service delivery are needed to achieve the goals outlined in the EHE strategy to prevent new HIV infections. A scoping review of 33 PrEP delivery models showed that centralized clinical settings (e.g., clinics, hospitals) were the primary site for PrEP and that PrEP was mostly provided by licensed prescribing clinicians and required “in-person” visits. This review, however, pointed to the need for innovations in service delivery, including telehealth and the use of community outreach workers, to reduce demands on patients and providers [26]. One such untapped option, however, is the co-prescribing of PrEP and MOUD in a low-threshold program utilizing APCs (such as APRNs). There are now a number of pilot studies documenting the use of APCs to deliver PrEP and MOUD (separately) with evidence of optimizing prevention services compared to the traditional model [29–32]. Yet, these low-barrier programs are destined to face challenges without structural changes, including simplifying the prescribing capabilities of APCs or expanding access to Medicaid benefits [33, 34]. Further advocacy for policy change pertinent to MOUD and PrEP is crucial to improving access for the most hard-to-reach populations.

Further coordinated care between relevant parties is needed to decrease demands on the overall system. From the implementation science perspective, automated and streamlined evaluations, modifying the order of evaluation, bundling and reflex testing, and standardized templates in the EMR are effective strategies to streamline services [35–37]. Streamlining services will also address barriers related to SDOH, such as an individual’s ability to pay for medications (e.g., coordination between health insurance and billing), their time interacting with clinicians, which can perpetuate stigma [38], or their access to transportation. For example, reducing in-person visits, especially telehealth without video, can overcome logistical and stigma-related concerns. Also, if follow-up support could be provided by case managers or counseling staff, it could facilitate an integrated team approach that increases comfort between patients and a diverse treatment team that could support retention efforts. Non-prescriber team members could have several checklists within their EMR, including clinical templates and ‘smart phrases’ to streamline eligibility assessment, medication readiness, and patient counseling. Such implementation tools, if used consistently, could increase providers' efficiency while simultaneously decreasing patient demands (e.g., reduced appointment times and fewer checkpoints in the care continuum), which can help facilitate integrated HIV prevention.

By identifying the breadth of concerns and the rank-ordered prioritization of solutions, the results of this study will inform the development of an integrated HIV prevention program that combines same-day PrEP and MOUD for PWID. The proposed solutions by PWID to be incorporated will minimally include home delivery of the medications by pharmacies, more accessible providers [e.g., through telehealth (phone) and telemedicine (video)], and increased access to transportation through the distribution of bus passes. The program will also include case managers, smart phrases and clinical checklists in the EMR, and designated appointment slots and time allocated for these visits to address the challenges posed by clinical stakeholders. Increasing the number of providers available to prescribe PrEP and MOUD (through decentralization) and adapting a low-threshold approach to improve accessibility (such as incorporating this program into ATCs or SSPs) will be necessary to consider when thinking about the future implementation and scale-up of this program.

In the implementation science field, we are often concerned with offering the “kitchen sink,” or the idea that our interventions are too multipronged to be effective in practice [39]. Findings from this study indicate the importance of offering a ‘one-stop-shop’ model of service delivery, considering patient preferences. For example, the future scalability of integrated, same-day HIV prevention programs will need to consider the ability to comprehensively address the social determinants of health through access to transportation, telehealth, and pharmacy delivery, among others. Though not all components might be a driver of success, having an array of options for participants is paramount. Future directions beyond the establishment of efficacy through a pilot-randomized trial could include the assessment of this program using the multiphase optimization strategy (MOST) [40]. MOST could help identify the critical components of the program that are minimally sufficient to address the complex needs of PWID accessing same-day PrEP and MOUD.

### Limitations and strengths

Although the NGT sessions were conducted to ascertain barriers and solutions to implementing a combined PrEP and MOUD program, the emerging ideas may be used to address generalized challenges in the PrEP or MOUD care cascade in the USA. Barriers such as lack of access (e.g., transportation, health care providers) are recurrent themes throughout the literature, highlighting one of the many ways this analysis mimics the current landscape; however, we also provide additional perspectives that may be used to confront issues in the standard of care [22, 23], which often places undue demands on patients. For example, our analysis articulated how we can solve challenges related to systems processing and expedited coordination that have not been noted elsewhere. On the other hand, the depth of our results was potentially limited due to the number of participants we could gather for both stakeholder groups. Because of our convenience sampling strategy, we did not comprehensively match the diversity of our catchment area. Though our sample was not optimally diverse regarding race or ethnicity, we could capture participants that likely experience a substantial burden of the social determinants of health through other mediated factors like education and socioeconomic status. Despite the small sample size, we could triangulate results across discussions, evidenced by the synergies in responses seen over the five sessions. Additionally, our approach allowed us to speak to both community (i.e., PWID) and clinical stakeholders. This method provided a unique perspective on a combined PrEP and MOUD process and empowered us to make decisions that will positively impact future interventions. In this process, we recognize that separating PWID and clinical stakeholders may have prevented learning and sharing of ideas across groups. We also recognize the overall limitations in the design of our discussions which limited our ability to capture innovations in the base design of our program. For example, discussions of different prescribing strategies to achieve the same goal were not explored to address the potential lack of feasibility in same-day PrEP plus MOUD. The NGT enabled us to take a multidimensional approach to largely qualitative themes, which allowed us to elicit issues related to the program and the current care cascade and rank them concerning significance. Ranking versus thematic analysis ultimately provided us with the tools necessary to prioritize solutions deemed most valuable by PWID and clinical stakeholders for implementation purposes.

## Conclusions

In the USA and elsewhere, HIV prevention efforts among PWID have been thwarted by multiple factors. Integration of PrEP and MOUD services with the adaptation of a low-threshold approach to improve accessibility (such as incorporating this program into ATCs or SSPs) will be necessary for “getting to zero” HIV infections among PWID. Same-day prescribing of PrEP and MOUD is an emerging implementation strategy to address the recent declines in HIV prevention in PWID. This study articulated the need for an integrated care model for same-day PrEP and MOUD delivery, which will be essential for future population-level scale-up. Including additional components to this model, such as transportation vouchers, can complement and increase the feasibility of this type of program. These findings on barriers and facilitators to same-day access to PrEP and MOUD will be important to inform the scale-up of new HIV prevention strategies in our specific target population, health care system, and social context. Additionally, with increased funding for harm reduction, this study highlights some necessary components that should become embedded opportunities for SSP clients, including regular and cost-effective access to transportation, telehealth, and home delivery of medications.

## Supplementary Information

**Additional file 1:** NGT focus group guide.

## Data Availability

The datasets used and/or analyzed during the current study are available from the corresponding author upon reasonable request.

## Abbreviations

APCs: Advanced practice clinicians
ART: Antriretroviral therapy
ATC: Addiction treatment center
CDC: Centers for Disease Control and Prevention
DEA: Drug Enforcement Agency
EHE: End the HIV epidemic
EMR: Electronic medical record
FQHC: Federally qualified health centers
MOUD: Medications for opioid use disorder
NGT: Nominal group technique
OUD: Opioid use disorder
PrEP: Pre-exposure prophylaxis
PWID: People who inject drugs
SET: Screen, evaluate, and treat
SSP: Syringe services program
TasP: Treatment as prevention
US DHHS: U.S. Department of Health and Human Services
