# Supplementary material for: Using nominal group technique to identify barriers and facilitators to preventing HIV using combination same-day pre-exposure prophylaxis and medications for opioid use disorder
Source: Harm Reduct J. 2022 Oct 28;19:120. doi: 10.1186/s12954-022-00703-8 (PMC9616614; doi:10.1186/s12954-022-00703-8)
Supplement: Supplementary file 1 — Additional file 1: NGT focus group guide. [file 12954_2022_703_MOESM1_ESM.docx]

**NGT Focus Group for the iRaPID Study**

*Applicable to all groups that include* ***PWID***

a) Opening Statement and confidentiality:

(Session leader introduces themselves and the research staff present in the meeting)

I would like to begin by thanking each one of you for attending today’s session. I welcome you to this meeting and want you to know that I am delighted to have each of you with us today. Our task today is very important and I believe that hand in hand, we will be able to go through with it. During this meeting you will help us design a program that we believe is going to help many people throughout the community.

It is called iRaPID and it literally stands for the integrated rapid access to an HIV prevention program for people who inject drugs. It is a program that will allow you to get two kinds of medication at the same time. This will allow us to protect people who inject drugs against HIV and to treat their opioid use disorder at the same time, in a more rapid and convenient way.

The success of this activity will depend on the equal and full participation of all members. I cannot stress how important it is for every participant to fully share their ideas, but also to allow others to participate as well. We are very excited to have the opportunity to talk to you all, as you all have important insights as to how we can improve the design of our project and eventually help those who might need it.

Some of you may know each other and some may not. Whether you know each other or not, we ask that each of you make a verbal commitment to keep the information we share today confidential. To help protect your confidentiality, please use pseudonyms and/or point and make eye contact at another individual if you wish to refer to them. Let us take a moment now for you to tell us if you have any concerns about being able to keep others’ information and disclosures confidential.

**Sessions leader waits and addresses all concerns (if any)**

Are there any questions before we proceed?

b) Rules, regulations, and preparation to start:

During the session, we will be asking questions one at a time and giving you time to come up with possible answers, followed by writing them down. Please read the document we are handing out right now carefully *(see appendix A).* It explains the process we will be following throughout the session and some rules that you will have to follow.

**hand out Appendix A** **wait a few minutes* *leader may choose to go over the sheet with the participants**

The session will be audio-recorded, and a transcript will be made to facilitate analysis of the information and opinions expressed. Are there any questions before we begin? I am going to start the recording. ********Start recording********

c) Questions:

Preamble: Thank you for agreeing to help us understand how to best get you treatment and prevention. Our goal is to be able to help you out. Before we get to our main questions, we would like to define a few things to make sure we are all on the same page. The two things are PrEP and medications for opioid use disorder or MOUD. As I said earlier, we will be trying to make a program that would allow us to give both kinds of medication at the same time.

1. PrEP stands for Pre-Exposure Prophylaxis and refers to a pill that you can take daily that can help prevent you from getting infected with HIV, especially if you share syringes or any injection materials. It will also protect you from getting HIV from sex. Is there anyone who is not familiar with PrEP? Let me know if you have any questions or if you would like me to explain this further.
2. Let us move to medications for opioid use disorder or MOUD. These are medications that are used to effectively treat opioid use disorder. They are either buprenorphine, which your regular doctor can prescribe if they have completed a short training, or methadone which must be given daily at a specialty treatment program. These medications treat opioid use disorder, reduce opioid use, and prevent HIV transmission by over 50% and Hepatitis C Virus transmission by over 60%. Is there anyone who is not familiar with methadone or buprenorphine? Again, let me know if you have any questions regarding this or if you would like me to repeat.
3. Now, what we need you to help us with is trying to understand the issues, as you see it, about getting both medications – PrEP **and** medications for opioid use disorder or MOUD – at the same time, even on the same day.

Now what I would like to do is pose a question and after you think about it for a few minutes, start to make a list of what you think. Your list does not need to be the same as anyone else. What we hope to do is generate the maximum number of ideas so we can talk more about them.

Question #1: If a person wanted to start on PrEP and MOUD (like methadone or buprenorphine), what kinds of things might get in the way of getting it prescribed to them on the same day?

Now, let us take 2 minutes and let you think about and make your own personal list of things that might get in the way …. There is a pencil and paper for you to use.

Now I would like to have each of you share your ideas with the other members of the group. If any one of you does not have any new ideas, you can pass your turn. [Turning to the first person in the group, ask:] Would you please give me one idea from your list?

**DO THE RANK ORDERING ON THE WHITE BOARD**

Question #2: Based on the top three priorities identified in the last question [listed for review], what types of resources or support do you think are required for developing a program that provided both PrEP and MOUD on the same day?

**FOLLOW SAME PROCESS FOR QUESTION #1**

DISCUSS RANKING

d) Ending Statement and Thank You Note:

This marks the end of our session. I would like to thank you all one more time for your participation today. You have helped us significantly and we are very grateful for all your efforts. Have a nice day! **Stop recording**

*Appendix A*

Rules and Regulations for Focus Groups

A) Session Guide:

**Step 1: Writing Down Answers**

Describe each idea in a brief phrase or a few words on the paper in front of you. Please work independently of other members. During this period of independent thinking, do not talk to other members, interrupt their thinking, or look at their worksheets. Since this is an opportunity for each of us to prepare his or her contributions to the meeting, we would appreciate intense effort during that time.

**Step 2: Sharing Ideas**

We will go around the table multiple times and ask each member, one at a time, to give one idea from their worksheet, summarized in a brief phrase or a few words. If someone else in the group lists an idea which you also had on your worksheet, you need not repeat the idea. If, however, in your judgment the idea on your worksheet contains a different emphasis or variation, we would welcome the idea.

**Step 3: Serial Discussion**

The purpose of this discussion is to clarify the meaning of each item on our flip chart. It is also the opportunity to express our understanding of the logic behind the idea and the relative importance of the item. The creator of the idea being discussed need not feel obliged to clarify or explain it. Any member of the group can play that role.

**Step 4: Voting and Ranking**

You will be provided with stickers to be able to vote (express your opinion). We will ask each one of you to come up and place stickers next to the ideas you think are most important. You can put all your stickers next to one item if you feel very strongly about the item, or you can put one sticker next to three items, or two stickers and then one. We will rank the top votes and discuss them.

B) Rules and more:

1- Please refrain from using your cell phone during this discussion and please switch it to silent mode.

2- Please try to protect each other’s confidentiality. Some of you may know each other. Even if you do, please respect each other’s privacy and confidentiality by not mentioning each other’s presence in the group to other people.

3- Please respect each other and each other’s opinions. There is no incorrect way to feel or think here and we want to encourage everyone to have the opportunity to share their thoughts. We do, however, want you to interact with and respond to each other to see how opinions are the same or different.

4- Please try to speak one at a time, so we can listen to what others have to say. This will also make it easier to transcribe our discussions accurately.

Thank you and good luck! ☺
